# “Using theories” and “doing theory”: tool and action orientations to theory at the interface of public health and anthropology

**DOI:** 10.3389/fpubh.2026.1707743

**Published:** 2026-04-30

**Authors:** Peter Taber, Eileen M. Dryden, Jean M. Hunleth, Linda S. Kahn, Lance Laird, Teresa Winstead, Genevra F. Murray

**Affiliations:** 1 Department of Biomedical Informatics, Eccles School of Medicine, University of Utah, Salt Lake City, UT, United States; 2 Center for Health Optimization and Implementation Research, VA Bedford Healthcare System, Bedford, MA, United States; 3 Department of Population and Quantitative Health Sciences, T. H. Chan School of Medicine, University of Massachusetts, Worcester, MA, United States; 4 Department of Surgery, WashU School of Medicine, Washington University, St. Louis, MO, United States; 5 Department of Family Medicine, Jacobs School of Medicine and Biomedical Sciences, University at Buffalo, Buffalo, NY, United States; 6 Department of Family Medicine, Chobanian and Avedisian School of Medicine, Boston University, Boston, MA, United States; 7 Department of Psychiatry and Behavioral Sciences, School of Medicine, University of Washington, Seattle, WA, United States; 8 Department of Public Health Policy and Management, School of Global Public Health, New York University, New York, NY, United States

**Keywords:** anthropology, epistemology, social science, team science, theory

## Abstract

The use of “theory” is a common concern in public health and anthropology. However, the term can have quite distinct meanings. In this piece, we seek to name and characterize some tendencies in anthropological theorizing that contribute to potential misunderstandings when communicating with non-anthropologist colleagues in public health. In particular, we highlight differences between the use of recognizable, named “theories” that often explicitly frame public health research and interventions, and the open-ended and context-specific work of “doing theory” sometimes referenced in anthropology. Observing the linguistic shifts entailed to refer to theory in each of these cases—“using theories” versus “doing theory”—we differentiate between “tool” and “action” orientations to theory. We then examine three case vignettes of engagements with “tool” orientations to institutionalized theories, and with “action” orientations to context-tailored theorizing in real-world public health interventions, showing how anthropologists have navigated these orientations. We close by emphasizing the benefits of epistemic diversity for interdisciplinary public health research and interventions.

## Introduction

Debates about the need for, and uses of “theory” are a recurring feature of both public health and anthropology, the home discipline of this piece’s authors. Within public health, these engagements can take the form of guidance and advocacy for increased use of theory (1, 2), inquiries into the prevalence and effectiveness of specific theories (3, 4), calls for applications of theory in specific domains (5, 6), and even the proffering of “integrated” or “unified” theories of specific phenomena (7, 8). Here, we argue that a shared concern with something called “theory” tends to mask differences in how anthropologists and our collaborators in public health characteristically engage with theory, and what we expect it to help us achieve in our shared work. In naming and characterizing divergent ways of engaging with theory at the interface of public health and anthropology, we hope to improve the ability of practitioners—including ourselves—to leverage these orientations to address real-world public health problems.

To begin, we note that public health, as well as some public health-facing anthropology, share an interest in, and assumptions about “theories”: that they offer discrete, coherent systems of concepts to describe the world and support research question- or hypothesis-generation, causal explanation and prediction. We contrast this with the work of “doing theory” within academic anthropology. While anthropology embraces diverse approaches to theory, these approaches are generally marked by the importance of ethnography—the firsthand, longitudinal and immersive study of a people, place or problem via a multi-modal approach that may include qualitative and quantitative methods—as academic anthropology’s principle approach. Consequently, anthropological theorizing tends to emphasize the ongoing incorporation of inductively discovered insights and context-specificity. In our experience, ethnography’s import to anthropology inflects anthropologists’ orientations toward theory even when ethnography is not a part of their immediate efforts, as is often the case in public health research and interventions.

Additionally, since at least the mid-1980s, anthropology’s emphasis on ethnography has been explicitly coupled with a disciplinary insistence on denaturalizing and critically examining received institutional categories and knowledge (including those used to formulate and enact public health interventions) as these play out in everyday experience. We suggest that both these historical factors—a methodological focus on ethnography and a philosophical orientation toward the critique of institutional power—contribute to the contemporary expectation that anthropological theory should be fluid and highly situationally tailored.

This manuscript emerges from the authors’ experiences of challenges thinking and communicating about theory with non-anthropologist public health practitioners. In our conversation around these challenges, we have observed a consistent grammatical shift: from *using theories*—named and internally coherent systems that might frame a workshop or grant applications—to *doing theory* by weaving together disparate views and understandings that arise in the course of empirical research. To reflect this linguistic shift, we distinguish in this piece between “tool” and “action” orientations to theory. In this distinction, we see the “tool” orientation approaching theory as something that has already been fashioned and is readily available for use; the “action” orientation to theory treats it as something enacted via the research process, requiring research and reflection over time to arrive at ideas properly fitted to a group of people, problem and/or context.

In Part 1, we seek to more fully explain our proposed distinction between “tool” and “action” orientations to theory via contrastive examples taken from public health and academic anthropology, respectively. In Part 2, we examine three authors’ experiences navigating tool and action orientations to theory in public health via case vignettes, highlighting some differences and similarities across cases. In Part 3, we close by re-emphasizing that “tool” and “action” orientations to theory are not mutually exclusive, and that different interventions, settings and research phases may call for different combinations and emphases. Ultimately, we argue that recognizing diversity in our orientations to theory improves our chances of using their tensions and synergies productively.

## Part 1: “Tool” and “action” orientations to theory in public health and anthropology

To justify the distinction between “tool” and “action” orientations to theory, we discuss two highly contrastive examples of theory relevant to both public health and anthropology. We first examine a statement by Karen Glanz and Donald Bishop on the role of behavioral science in public health in a review of the field (1), and then turn to a description of ethnographic theory by medical anthropologist João Biehl (9).

*Using theory: The “tool orientation” to theory*. Glanz and Bishop define “theories” as sets of “interrelated concepts, definitions, and propositions that explain or predict events or situations by specifying relations among variables. The notion of generality, or broad application, is important. Thus, theories are, by their nature, abstract and not content- or topic-specific” [401]. The authors emphasize complexity and systemic connectedness for adequate theories in public health interventions: “Many social, cultural, and economic factors contribute to the development, maintenance, and change of health behavior patterns… Families, social relationships, socioeconomic status, culture, and geography are other important influences. A broad understanding of some of the context, key factors and models for understanding behaviors and behavior change can provide a foundation for well-informed public health programs…” [400].

Theories as described by Glanz and Bishop and others (10, 11) offer a systemic picture of how the world works in a particular domain (description), and insight into the drivers or determinants that contribute to public health problems of interest (causal explanation). Glanz and Bishop seem to have in mind theories commonly used in tandem with deductive research designs and quantitative methods, and that can facilitate probabilistic prediction. Yet, we suggest that much of their vision of theory could also be extended to theoretical traditions used in humanistic research, to the extent that those traditions aspire to produce theory that is generalizable, systemic, internally coherent, and self-contained.

Glanz and Bishop evince a “tool” orientation to theory: a theory as an individuated, already-formed system comprised of internally coherent concepts. Under this definition of a theory, it makes sense to ask which theory is guiding a public health intervention, or to gauge the merits of one theory versus another for a given public health domain in terms of their respective evidence bases or effectiveness. This version of “theory” is likely concordant with many people’s common-sense understanding of the term.

*Doing theory: The “action orientation” to theory.* Contrast the above orientation with a statement by medical anthropologist João Biehl (9) on the ethnographic theory that he believes is demanded by anthropology’s engagements with global public health:

“Ethnographic theory emerges from and in conversation with people and world-making practices, with various ways of knowing and relating. It is a way of staying connected to open-ended social processes and unknowns—a way of counterbalancing the generation of certainties and foreclosures by other disciplines…” [136].

Biehl stops short of providing a definition of ethnographic theory, but responsiveness to contingency and context seem key in his view. The emphasis in this passage on local specificity and the provisionality of theory in a given case suggests that theory’s generalizability is not Biehl’s focus. In contrast with Glanz and Bishop, Biehl’s writing emphasizes theory as something requiring ongoing action in the course of research (9). Theory here is open-ended and built longitudinally, by iteratively retooling a project’s conceptual framing. In Biehl’s view, we are less likely to arrive at adequate theory by selecting whole conceptual systems from an enumerable set of *a priori* options, and more likely to build it by emphasizing process, bringing together concepts that respond to the evolving details of research. From this perspective, the very question of “which theory” a scholar should select in advance to guide their research would reflect a misunderstanding of how to conduct research.

Tool and action orientations to theory differ most prominently in two respects: first, their respective goals of causal explanation vs. context-driven critical attention to power differentials, their reproduction, and their real-world effects; and second, the degree to which they rely upon recognizable, institutionalized and named systems of concepts for analysis. They also differ in terms of expectations about the self-contained nature of theory; whether theory can be selected *a priori* or must be iteratively developed through research; expectations about generalizability; and the intended utility of theory to public health interventions. Additional points of divergence could likely be identified through the consideration of more examples.

The proposed tool/action heuristic is not a conventional way of discussing theory in anthropology, but one that we believe usefully highlights complex differences that are otherwise tacit and difficult to identify. We use the heuristic primarily to label *orientations to theory*, rather than *types of theory*. For example, an action orientation to theory might engage with a specific, established, theory to extract situationally useful constructs, whereas a tool orientation might be inclined to use the same theory to frame a project, expecting fidelity to that framing throughout the life cycle of the project. Moreover, as one reviewer noted, anthropology is replete with established theoretical paradigms that offer relatively coherent and systematic frameworks (structuralism, functionalism, Marxist approaches), which could be taken as ready-made tools for framing research. Thus, there is a spectrum of orientations to theory at play, from tool to action, even within the discipline. Finally, while we emphasize the subjective orientation to theory (what we normatively think theory should consist of and how we should engage with it), the intellectual products resulting from these orientations seem likely to be linked: a tool orientation may lend itself toward building coherent systems, while an action orientation may encourage more context-specific and critical conceptual contributions.

In our experience, the tool orientation to theories as individuated, descriptive/causal-explanatory models tends to be the assumed or explicit orientation in the discourse of major funding agencies like the U.S. National Institutes of Health (NIH). In contrast, within anthropology, some have observed that “theory” now serves as a gloss for diverse ways of thinking about and conducting anthropological practice that tend to privilege provisionality and context-specificity (of which Biehl’s is only one example) (12). Many funders, including the NIH, have taken significant steps to incentivize team science approaches in which both of these orientations to theory are likely to be present. Moreover, adapting to context is increasingly invoked by public health researchers, as well as NIH and other funders, as critical to the success of health interventions (13–15); an action orientation may be important for addressing challenges facing current definitions and approaches to context. To explore the interplay of tool and action orientations to theory in practice, we now turn to examples of real-world interdisciplinary collaborations.

## Part 2: The interplay of tool and action orientations to theory in anthropological engagements with public health

Thus far, we have indicated two contrastive tendencies in how anthropologists and non-anthropologists orient to theory in public health and anthropology. In this section, we offer three examples from our own work in public health to highlight different ways of interweaving tool and action orientations to theory in practice. The first example, from Linda S. Kahn, shows how an anthropologist PI approaches an established implementation science theory to be more responsive to the individuals and communities in question. The second example, from Lance Laird, examines team research that includes anthropologists in non-PI roles, with data collection and initial analysis predicated on a tool orientation to theory, paralleled by a qualitative study arm drawing upon situationally appropriate constructs from academic anthropology to understand unexpected findings. The final example, from Jean M. Hunleth, shows how an anthropologist-headed team works with an action orientation to theory to foreground children’s agency and enable an alternative kind of interdisciplinary public health teamwork in the context of a vaccination program implementation.

### Vignette A: Normalization process theory (NPT) with an anthropological twist (Linda S. Kahn)

Anthropologists working in public health may find themselves integrating action orientation to theory, including inductive thinking and iterative interpretation of the meaning making from close analysis of participant data, even within a more tool-oriented context. For example, NPT is a widely used implementation science theory that elucidates how new processes and practices are operationalized and sustained in healthcare and other settings (16). NPT entails four interacting constructs, including sense-making (understanding the meaning and value of a new practice), cognitive participation (individual engagement in a new practice), collective action (coordinated efforts of individuals and groups to operationalize a new practice), and reflexive monitoring (evaluating and adapting a new practice based on feedback) (16).

As an anthropologist, I (LSK) have engaged with NPT within and outside health care institutions (17, 18). Using an anthropological toolkit shaped by an action orientation that emphasizes process, experience, and the critical role of narrative, I ask open-ended questions to elicit people’s stories about their reactions to new situations, the meanings they ascribe to these experiences, and how they incorporate new practices. I allow the participants’ voices and conversations to provide context and inform my interaction with normalization process theory. NPT has helped my team to reflect deeply about how people and organizations adapt to change, as we analyze our observations and findings in relation to NPT constructs.

We used NPT blended with anthropology to explore low-income patients’ experiences with early-stage chronic kidney disease (CKD) (18) We learned that implementing, and “normalizing” new procedures or behaviors is neither linear nor uniform, and is often distinct to each person. Patients in our study had limited knowledge of CKD, and some expressed confusion about being diagnosed with this “silent disease” in its earliest stages. During home interviews, patients described their challenges managing multiple chronic illnesses, occasionally bringing out bags of medications or trays laden with pill bottles. Patients frequently mentioned the importance of church, their pastor, and faith in God to their chronic illness self-management and coping strategies. Social drivers of health, especially transportation and financial constraints, posed barriers across NPT constructs, affecting patients’ abilities to incorporate CKD and other chronic illness self-management routines.

Recently, May et al. distilled NPT into a comprehensive qualitative coding manual to promote generalizability and simplify the application of NPT for qualitative researchers (19). While the authors are careful to explain that the manual is only intended as a helpful guide and acknowledge that qualitative coding is only part of a larger process of theory-informed qualitative work (19), as an anthropologist I am most comfortable allowing the data to speak for themselves and then reflecting on how our findings correspond to NPT, rather than limiting my scope with a coding manual. In this way, I balance the strengths of a tool orientation to NPT, against the generative potential of an action orientation to contextualized anthropological theorizing. The process of integrating anthropology and NPT can provoke questions about the diverse ways people and organizations react and adapt to change, while leaving space for inductive reasoning, inspiration, and surprise.

### Vignette B: Sensitizing a U.S. dental care intervention to Somali refugee oral health practices (Lance Laird)

Anthropologists may use an action orientation to critique and bring nuance to larger projects in which they are embedded, which may be framed by clearly defined, pre-existing frameworks via a tool orientation to theory. For example, a multidisciplinary oral health study with Somali refugees tested relationships between acculturation, English language proficiency, health literacy, and dental health. Study design was guided by the Health Belief Model (20), an established health theory that posits a rational actor evaluating the benefits of, and barriers to, preventive measures. The study used psychological and behavioral measures of acculturation (e.g., language proficiency, media use, social interactions, food preferences, and ethnic self-identification) (21), as well as a health literacy test (22). Investigators hypothesized that acculturation may have both positive and negative health effects, but that English language proficiency and health literacy would result in lower levels of decayed, missing and filled teeth (DMFT).

The team surveyed 439 adult Somalis who had lived in the United States 10 years or less, conducted examinations to count DMFT, and measured English proficiency and health literacy. Contrary to the study’s initial hypotheses, we found that literacy was not significantly associated with DMFT. Somali refugees who had been in the U.S. for less than 4 years had generally good oral health status and dental hygiene, although only 36% had seen a dentist for preventive care in the prior year. Those who had resided in the US for 5–10 years had higher rates of DMFT, with lower health literacy marginally correlated with this finding (23).

In the study’s qualitative arm, my anthropology colleague and I (LL), a medical anthropologist specializing in U.S. Muslim communities, interviewed 83 participants to explore how recently arrived Somali refugees understand and make decisions around their oral health. With an action orientation to theory, topics were drawn from the Eraker Health Decision-Making Model to describe individual knowledge, past experiences with health professionals, methods of health maintenance, and attitudes toward care (24). We added questions from social models of decision-making (how individuals often learn from others in their community) about how participants learned to care for their teeth and treat oral pain as children. Finally, to understand how refugees’ social worlds were transformed in camps and resettlement sites, we also drew on ethnomedical theories that recognize multiple, dynamic, cultural frameworks (the biomedical/dental system being only one) for labeling illness, preventing or treating suffering, and choosing among different types of healers (25, 26).

We found that Somali Muslims frequently used a “stick toothbrush” (*aday* or *miswak*) from the *Salvadora persica* tree in preparing for daily prayers. Following anthropological theory concerning the “social life of things” (27) (e.g., products like pharmaceuticals and devices), we explored toothbrushing as a religious and health obligation in transnational movements (28).

Contra the “deficiency discourse” common in some domains of public health, we advised cultural humility among dental professionals, recognizing migrants’ health assets and emphasizing “the integration of oral health practices common in the U.S. into Somali traditional and religious practices” (29). We concluded that for U.S. Somali refugees “oral health care remains rooted in Islam. By acknowledging the value of traditional practices, dentists may communicate the value of Western preventive and restorative dentistry, and recommend approaches to integrating the two” (29). In sum, we mobilized an action orientation to theory to foster richer analysis (the social life of things) but in order to make a recommendation that would make sense to our clinical colleagues, our findings addressed the institutionally recognized theory embodied in the overarching project (cultural humility).

### Vignette C: The reaching for equity in adolescent care study (REACH): Theory as everyday practice (Jean M. Hunleth)

An action orientation to theory from the outset of a project or collaboration can be used to foster more equitable relations within a research team and bolster forms of agency that institutionalized approaches to public health might overlook. Zambia aims to achieve the World Health Organization’s goal to have 90% of girls vaccinated against HPV by age 15 (in 2030). The Reaching for Equity in Adolescent Health through HPV Vaccination (REACH) study is a 5-year National Cancer Institute-funded study to increase HPV vaccination access and uptake in Zambia, a collaboration between myself (an anthropologist), a Zambian pediatrician-researcher, and colleagues from medicine, epidemiology, and implementation science (30). The idea for our collaboration started with a cellphone video, taken at a school during an HPV vaccination campaign. The video showed girls jumping out of classroom windows to escape the nurses who had come to vaccinate them.

I (JMH) am an anthropologist of childhood who studies global health programs and child health policies in Zambia and globally. In my prior work, I have critiqued such programs and policies as driven by visions of children as passive, in need of protection, uninformed, irrational, and incomplete (31–33), a view that took root in enlightenment and colonial conceptions of childhood. HPV vaccination programs follow similar patterns, with much programmatic focus on parental hesitancy and consent, and limited attention to children (34). As a PI of this study, I did not want to play into the invisibilizing of children’s concerns, needs, and wishes. The girls’ overt resistance to vaccination spoke to the limits of such an approach. This is where the anthropology of childhood holds theoretical value to advancing global health policy and programming (35). The anthropology of childhood aims to identify and decenter adult-centric approaches to childhood by taking an action orientation to incorporate theories of power, agency, and interdependence (36).

Most fundamentally, such theory considers children as full human beings. While seemingly obvious, this theoretical orientation requires a substantial shift in thinking for most adults. To do that work, we built theory into our daily practice, through trainings and readings related to power dynamics and structural inequalities, and through reflexively attending to our adult biases. Such practices are necessary if we are to avoid being complicit in producing strategies that further oppress children (37), and our findings showed the importance. Some things we learned from the 93 girls who participated (that we did not learn from the 132 participating adults) included: the responsibilities placed upon girls within vaccination campaigns due to resource shortages and monitoring gaps (e.g., they were asked to remember their vaccine schedule); the dismissal of their concerns and needs within vaccination efforts that often happened with little advance notice; and the range of tactics girls used to get or avoid the vaccine (e.g., changing a consent form, lying about their age).

Childhood theory as everyday practice that interrogates power and privilege also shapes how I lead a 15+ person team of interdisciplinary researchers across Zambia and the United States. Because we do not relegate theory to something only certain people do (while others collect data), the production of knowledge has become more dispersed and co-produced (38). This, in turn, is part of undoing oppressive power structures in global health team research (39). Doing theory in this way also enables us to more collectively as a global team move toward identifying and adapting the strategies that we will use to integrate HPV vaccination into clinics. This work often requires the use of a tool orientation to theory, drawing on established frameworks that may be unfamiliar to people on the team who are not from certain backgrounds. However, acknowledging and undoing power dynamics that have concentrated theory as something that only certain people on teams ‘do’ allowed us to all learn, critically interrogate, and apply theories to inform the mechanisms of action of these strategies.

In Vignette A, LSK’s project utilized NPT, an established theory from implementation science with clearly defined, albeit broad and highly malleable, domains. NPT was interpreted in relation to the specific needs of the project via inductive qualitative research. LSK’s open-ended methods, characteristic of anthropology’s action orientation to theory, were guided by the anticipation that patients’ responses to a new diagnosis would be shaped by a complex mix of factors, including cultural values, resource constraints and health knowledge. LSK notes that capturing these factors required an intentionally open-ended approach that might have been impeded by standardized data collection instruments or analysis procedures, if they encouraged the research team to forego deep consideration of real-world lived experience.

In Vignette B, LL’s examination of the “social life” of the *aday/miswak* as it was utilized by a diasporic population highlighted the crucial role of culture in shaping health practices that could have been overlooked if the project had solely utilized an individual-focused tool orientation to theory. For example, exclusive reliance on the Health Belief Model does not account for health practices that people engage in for reasons other than avoiding sickness or pain (e.g., for appearance, as religious practice, or out of unconscious habit). Here, an action orientation to theory within the anthropologist-led project arm made sense of unexpected findings in the parent study by drawing on a concept from the anthropology of globalization, based on its fit with the cultural practices of a refugee population of interest.

In Vignette C, JMH’s project was framed in terms of childhood theory, an original perspective assembled to deal with the collaboration’s interests via an action orientation to theory, and with an explicit critique of widespread public health assumptions about the nature of childhood and agency. The project’s overall theoretical framing may have fit awkwardly into assumptions of funders or partner organizations derived from conventional public health tool-oriented theoretical framings, but took seriously the perspectives of children, yielding insights with direct consequences for a public health intervention. Moreover, this theoretical approach is not just about children but about interrogating power dynamics across career stage, researcher age and status, and disciplinary knowledge. Such an approach is critically needed in all public health research groups and especially in global public health work where teams are dispersed, and there exist institutionalized hierarchies among and between researchers situated in the Global North and South that can constrain knowledge production. The process-based and reflexive work of doing theory constituted a form of everyday practice that was integral to the management of the project and collaboration of the team. This, in turn, benefited the team as they co-developed implementation strategies, work that required drawing on and identifying useful pre-existing frameworks and theories.

Despite their many differences, across all the examples we observe three shared features. First, the examples evince differing degrees of emphasis in the action orientation to theory, and differing ways of integrating that orientation alongside a tool orientation. The examples thus help us to see that the two orientations are not mutually exclusive, although they are likely to serve different ends. Second, all three examples exhibit awareness of the importance of culture and society in shaping the individual. Often, this awareness is in tension with the methodological individualism that dominates in public health research, and broader Western assumptions about personhood. Third, the vignettes demonstrate the use of theory to devise research strategies capable of discovering complexity and diversity, pushing research beyond tacit assumptions, *a priori* concepts or institutionally-sanctioned models. As Biehl might have it, the vignettes demonstrate how the authors leveraged theory to “[counterbalance] the generation of certainties and foreclosures” of public health, while informing and improving real-world interventions.

## Part 3: Reflections on theories, theory and critique at the interface of public health and anthropology

While the vignettes above indicate diverse ways of navigating tool and action orientations to theory, they also demonstrate two key shared anthropological commitments: (i) a commitment to constructive critique of prevailing systems, power structures, and modes of thought; and (ii) an emphasis on deeply nuanced, inductive exploration of context as core to any empirical science of culture, society, and health. While these commitments are likely to play out through diverse research designs and methodologies in public health, we reemphasize the importance of ethnography’s imprint on the epistemology and sensibilities of the discipline, even when anthropologists are not directly engaged in ethnography.

We appreciate thinking, with Biehl, that tool and action orientations to theory in medical anthropology are in part informed by the discipline’s methodological commitment to holding multiple visions of the world in dialogue at once, opening space for complexity, tension, and critique in public health. Rather than seeing such tensions as always in need of resolution through scholarship, we take such tension itself as both inherent to the human state and highly generative. Fellow ethnographer Soyini Madison nudges this argument further along in relation to her own vision of critical theory, offering that when situated theoretical work, akin to our action orientation to theory, is combined with ethnography, they can amplify our contributions to equity. She argues that, “[c]ritical ethnography becomes the ‘doing’ or the ‘performance’ of critical theory. It is critical theory in action.” (40) What we term an action orientation to theory communicates a commitment to holistic, rigorous, continuous and methodologically responsive inquiry, and understands systems of power as paradigmatic (and slippery, often hidden in plain sight), necessitating a strategic commitment to responsive context-specific tools to understand social worlds as always evolving.

Anthropologists engaged in public health, and the authors of this article in particular, are often engaged in this kind of foundational critical theoretical work, even when an action orientation to theory is not clearly visible in their work. This often tacit critical component of anthropological theory is important to the contribution anthropologists make in public health research, transforming what may be perceived by some as the application of mere methods into an invitation to epistemological dialogue and progressive scientific practice (41). By making this heretofore tacit knowledge explicit to a diverse audience of public health scholars, we aim to lay the foundation for deeper, more productive anthropological engagement in interdisciplinary public health team science.

### Key points for reflection

- Public health often references “using theories,” where theories are understood as named, discrete, internally coherent systems for representing and explaining the world.

◦ We term this a “tool” orientation to theory

- Anthropology often references “doing theory,” understood as the act of bringing together diverse concepts to respond to context and emergent insights.

◦ We term this an “action” orientation to theory

- Tool and action orientations to theory often tacitly co-exist on public health projects that include anthropologists.
- Attending to both of these ways of engaging with theory allows us to:

◦ Identify and productively use the tensions between these different orientations.
◦ Advance equity as we work in tandem with research communities.
◦ Avoid being blind to that which our own theoretical position may not have primed us to see.
◦ Maintain openness and epistemic humility as we theorize.

## Data Availability

Publicly available datasets were analyzed in this study. This manuscript addresses theoretical concerns in previously published qualitative research. Further inquiries can be directed to the corresponding author.
